# Glow in the D-ARK: a new bioluminescent species of *Corallizoanthus* (Anthozoa: Zoantharia: Parazoanthidae) from southern Japan

**DOI:** 10.1098/rsos.250890

**Published:** 2025-11-05

**Authors:** Hiroki Kise, Manabu Bessho-Uehara, Kenta C. F. Kondo, Kiko Shimoji, Shohei Ito, Shinji Tsuchida, Yoshihiro Fujiwara, James D. Reimer

**Affiliations:** ^1^ Integrated Research Center for Nature Positive Technology, National Institute of Advanced Industrial Science and Technology Tsukuba, Tsukuba, Ibaraki, Japan; ^2^ Geological Survey of Japan, National Institute of Advanced Industrial Science and Technology, Tsukuba, Ibaraki 305-8567, Japan; ^3^ The Frontier Research Institute for Interdisciplinary Sciences, Tohoku University, Sendai, Miyagi 980-8578, Japan; ^4^ Graduate School of Life Sciences, Tohoku University, Sendai, Miyagi 980-8578, Japan; ^5^ Molecular Invertebrate Systematics and Ecology Laboratory, University of the Ryukyus, Nishihara, Okinawa 903-0213, Japan; ^6^ FullDepth Co., Ltd., Tsukuba, Ibaraki 305-0006, Japan; ^7^ Research Institute for Global Change, Japan Agency for Marine-Earth Science and Technology, Yokosuka, Kanagawa 237-0061, Japan; ^8^ Tropical Biosphere Research Center, University of the Ryukyus, Nishihara, Okinawa 903-0213, Japan

**Keywords:** deep sea, karst, phylogeny, mitochondrial genome, *Pleurocorallium*

## Abstract

Bioluminescence is a common phenomenon found in many marine environments and has evolved independently dozens of times across the Tree of Life. In Anthozoa, a single origin of bioluminescence in Octocorallia has been proposed, while the evolution of bioluminescence in Hexacorallia remains unclear due to incomplete taxon sampling. This study, based on recent deep-sea surveys in southern Japan, describes a new bioluminescent *Corallizoanthus* species that is epibiotic on Coralliidae octocoral species and also provides observations of bioluminescence activity and spectral data for the new species and another parazoanthid species. As bioluminescence in Hexacorallia has been primarily found in order Zoantharia, integrating bioluminescence data into zoantharian taxonomy would allow not only a new understanding of the evolution of Hexacorallia, but also insights into the ecological aspects of bioluminescence in deep-sea environments.

## 1. Introduction

Bioluminescence is a common phenomenon that has been observed across the globe, from shallow waters to the deep sea [1–7]. Bioluminescence has been reported in at least 11 phyla of metazoans, most of which are marine organisms, and reported in diverse environments from the poles to the tropics, suggesting its biological importance [1,4,7]. It is known that bioluminescence has evolved independently dozens of times across the Tree of Life, although studies of the evolutionary history of bioluminescence have focused on taxa for which robust phylogenetic trees are available [8–12].

Subphylum Anthozoa includes benthic cnidarians that contain ecologically important foundational species in various marine environments, and bioluminescence has been found in some anthozoans, including 32 Octocorallia genera and eight Hexacorallia genera [5,9,13]. DeLeo *et al*. [9] explored the evolutionary history of bioluminescence in anthozoans and hypothesized a single origin for bioluminescence in Octocorallia, while the evolution of bioluminescence in Hexacorallia remains unclear, as few bioluminescence records have been reported. A comprehensive inventory of luminescent species, particularly hexacorallians, is needed to better understand the evolutionary history of bioluminescence in Anthozoa.

In 2024, we explored the deep-sea benthic ecosystems around Minamidaito Island in southern Japan using a remotely operated vehicle (ROV) as part of the Deep-sea Archaic Refugia in Karst (D-ARK) project led by the Japan Agency for Marine-Earth Science and Technology (JAMSTEC). Minamidaito Island is an oceanic island approximately 360 km to the west of Okinawa-jima Island and is known to have a dramatic underwater topography characterized by steep and complex structures, preventing collection of data and specimens using traditional deep-sea benthic sampling methods and resulting in a lack of information on marine biodiversity in this area [14,15]. During the D-ARK surveys, we discovered an undescribed bioluminescent species of *Corallizoanthus* Reimer in Reimer, Nonaka, Sinniger and Iwase, 2008 [16] (Hexacorallia: Zoantharia: Parazoanthidae) living epibiotically on *Pleurocorallium* coral colonies at the entrances of several karst caves. Based on integrated approaches, we formally describe *Corallizoanthus aureus* sp. nov. Furthermore, we investigated bioluminescence activities and spectra of three zoantharian species, *Corallizoanthus aureus* sp. nov., *Churabana kuroshioae* Kise, Montenegro and Reimer, 2021 [17] and *Epizoanthus fatuus* (Schultze, 1860) [18], and report on the results herein.

## 2. Material and methods

### 2.1. Specimen collection

We observed numerous *Pleurocorallium* colonies around entrances to karst caves on Minamidaito Island during the D-ARK surveys (KM24-03 Leg 2 cruise and KM25-06C) in May 2024 and July 2025. Two *Pleurocorallium* colonies were collected from northwest off Minamidaito Island, one on 6 May 2024 and another on 16 July 2025, by the remotely operated vehicles *KM-ROV* and the *TriPodFinder2* (FullDepth Co., Ltd, Japan) attached to *KM-ROV* aboard the R/V *Kaimei* at 385 m depth. The specimens were observed to harbour numerous polyps of an undescribed bright yellow *Corallizoanthus* sp. Photographs of the specimens were taken *in situ* by the *KM-ROV* and *TriPodFinder2* for gross external morphological observation before collection. The specimens were subsampled for bioluminescence tests and kept in the seawater in a cold dark room at 4°C until use. Upon specimen retrieval, the specimens were relaxed with magnesium chloride and subsequently fixed in 10% seawater formalin with subsamples preserved in 99.5% ethanol. The specimens examined in this study have been deposited in the National Museum of Nature and Science, Tsukuba, Japan (NSMT Co-1915 and NSMT Co-1919). In addition, we examined two other zoantharian deep-sea specimens, of *Churabana kuroshioae* and *Epizoanthus fatuus*, collected from the Kita-Koho Seamount, Kyushu-Palau Ridge, Japan at depths of 622 m and 1869 m during the KM24-03 Leg 2 cruise in May 2024, to further investigate luminescence activities of Zoantharia.

### 2.2. Molecular analyses

DNA was isolated from ethanol-preserved tissue of the holotype with a spin-column DNeasy Blood and Tissue Extraction kit following the manufacturer’s instructions (Qiagen, Hilden, Germany). Extracted DNA was quantified using a Qubit dsDNA HS assay kit (ThermoFisher Scientific, Waltham, USA), and 58 ng was used as input into library preparation. We used the NEBNext Ultra II FS DNA Library Prep Kit with some modifications according to Quattrini *et al*. [19]. DNA libraries were quantified and assessed with a Qubit dsDNA HS assay and a Microchip Electrophoresis System (MultiNA, Shimadzu, Japan) with a DNA1000 Reagent Kit (Shimadzu). Sequencing was performed on an Illumina NextSeq 1000 to target 150 bp of 10−20 M pair-end reads according to Quattrini *et al*. [19]. The raw reads were filtered using Trimmomatic v. 0.39 [20] with default parameters. Filtered reads were *de novo* assembled with GetOrganelle v. 1.7.5 [21], which implemented SPAdes v. 3.6.2 genome assembler [22] with K-mer = 115. The mitochondrial genome annotation was performed with the MITOS webserver [23] and manually inspected and adjusted using Geneious Prime 2025 (https://www.geneious.com). The annotated mitochondrial genome was deposited in GenBank with the accession number PV987613. Sequences of Cox1 (mitochondrial cytochrome c oxidase subunit I), 12S rDNA (mitochondrial 12S ribosomal DNA) and 16S rDNA (mitochondrial 16S ribosomal DNA) were extracted from newly obtained mitochondria genome. We also obtained nuclear sequences, namely 18S rDNA (nuclear 18S ribosomal DNA), ITS rDNA (nuclear internal transcribed spacer region of ribosomal DNA) and 28S rDNA (nuclear 28S ribosomal DNA) sequences, following the methods of Kise *et al*. [17]. Bidirectional nuclear sequences were assembled and edited in Geneious Prime. Obtained nuclear sequences were deposited in GenBank with the accession numbers PV930384–PV930386. Multiple sequence alignments were performed with previously published Parazoanthidae sequences obtained from GenBank (electronic supplementary material, table S1) using MAFFT v. 7.110 [24] with the auto algorithm under default parameters. Epizoanthidae Delage & Hérouard [25] were selected as outgroups. Phylogenetic analyses were performed on the concatenated dataset using maximum likelihood (ML) and Bayesian inference (BI). ModelTest-NG v. 0.1.6 [26] and the Akaike information criterion were used to independently select the best-fitting model for each molecular marker for ML analyses. The best models for ML were listed in the electronic supplementary material, table S2. For ML analyses, RAxML-NG [27] was utilized under the default settings with 1000 bootstrap replicates. For BI analyses, ExaBayes 1.5.1 [28] with the GTR substitution model was performed. Four independent Markov chain Monte Carlo runs were run in parallel for 3 000 000 generations, sampling every 500 generations. Convergence was determined when the average standard deviation of split frequencies was below 0.01.

The DNA sequence alignment generated is available in the Alignment S1.

### 2.3. Morphological observations

External morphological characteristics were observed and dissected under a Stemi 305 microscope (Carl Zeiss, Oberkochen, Germany). In addition, *in situ* photographs were used for morphological observations. Internal morphological characters were examined by histological sections; 10−15 µm thickness serial sections were made with a microtome (Leica RM2125 RTS Leica Biosystems, Wetzlar, Germany) and stained with haematoxylin and eosin after desilication with 15–20% hydrofluoric acid for 18−24 h. Classification of marginal muscle shapes followed Swain *et al*. [29]. Cnidom analyses were conducted using undischarged nematocysts and spirocysts from tentacles, column, actinopharynx and mesenterial filaments using a Nikon Eclipse80i stereomicroscope (Nikon, Tokyo, Japan), and photographs were taken by a Nikon DS-Qi2 (Nikon, Tokyo, Japan). Cnidom sizes were measured using ImageJ v. 1.45s [30]. The reported frequencies were the relative amounts based on numbers from all slides in the cnidom analyses. Cnidom classification generally followed England [31] and Ryland & Lancaster [32] except for the treatment of basitrichs and microbasic b-mastigophores as in Kise *et al*. [33].

### 2.4. Bioluminescence observation

The capability of bioluminescence was tested on board on the same day that the specimens were collected. The specimens in the seawater were transferred from the cold room to a dark room at an ambient temperature of approximately 27°C. The specimens were mechanically stimulated by pinching with forceps and chemically stimulated by the addition of a few drops of 2.5 M potassium chloride (KCl). The bioluminescent behaviour was recorded by a digital camera SONY alpha 7 s III (Sony, Japan), with a 50 mm macro lens, SEL50M28 (Sony). In addition, bioluminescence was also tested *in situ*; living polyps of colonies were stimulated using the suction sampler of *TriPodFinder2* during dives, and the bioluminescent behaviour was recorded by a SONY FCB-ER8530 (Sony) on *TriPodFinder2*.

### 2.5. Measurement of bioluminescence spectra

The bioluminescence spectra of the three zoantharian species were measured by a spectrometer FLAME-T (OceanInsight, Lake Mary, FL, USA) with a 200 nm slit, grating #2 (200–850 nm) and an optic fibre. The specimens were stimulated by gently touching colonies with the end of the optic fibre in the dark, and the resulting bioluminescence spectrum was collected for 3000 ms. The data were exported and analysed using a custom R script. In brief, the light intensity at each wavelength was smoothed, normalized and averaged among four replicates to determine the peak wavelength.

## 3. Results

### 3.1. Taxonomic account

Order Zoantharia Rafinesque, 1815 [34]

Suborder Macrocnemina Haddon and Shackleton, 1891 [35]

Family Parazoanthidae Delage and Hérouard, 1901 [25]

Genus *Corallizoanthus* Reimer in Reimer, Nonaka, Sinniger and Iwase, 2008 [16]

Type species. *Corallizoanthus tsukaharai* Reimer in Reimer, Nonaka, Sinniger and Iwase, 2008 (type by monotypy) [16]

Diagnosis (changes in bold). Characterized by its association with living precious corals (Alcyonacea: Coralliidae), and unlike the gorgonian-associated genus *Savalia*, does not secrete its own scleroproteinaceous axis. Additionally, **polyps are normally solitary and sometimes connected by thin stolon** [16]. Encrustations to centre of mesoglea, sphincter muscle is cyclically transitional [29,36].

*Corallizoanthus aureus* sp. nov.

Figures 1–3 and 4.

**Figure 1.**
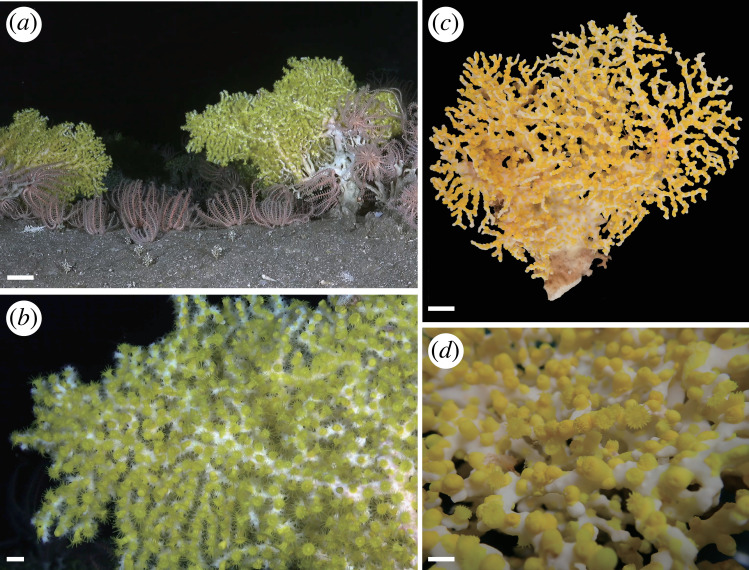
Images of external morphology of *Corallizoanthus aureus* sp. nov. *In situ* image (*a*), close-up *in situ* polyps (*b*), relaxed specimen (*c*) and close-up relaxed specimen (*d*).

**Figure 2.**
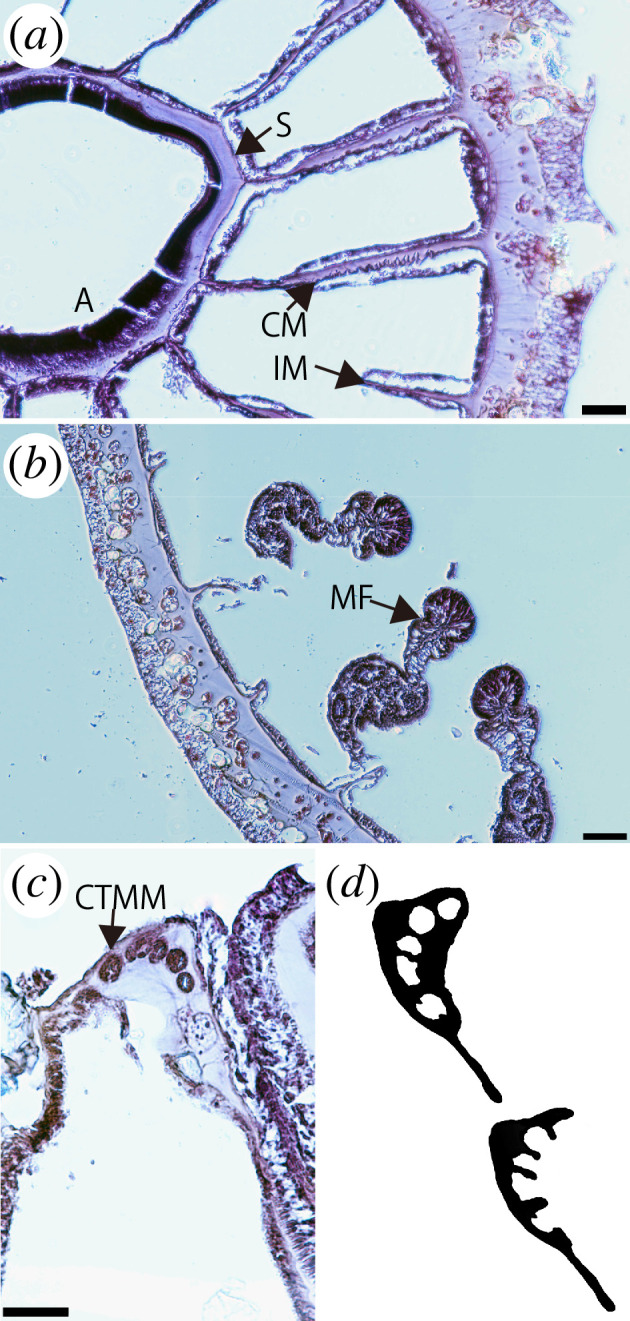
Images of internal morphology of *Corallizoanthus aureus* sp. nov. Transverse section at the level of the actinopharynx (*a*) and mesenterial filaments (*b*), close-up image of cyclically transitional marginal musculature (*c*), and illustration of cyclically transitional marginal musculature (*d*). A, actinopharynx; CTMM, cyclically transitional marginal musculature; CM, complete mesentery; IM, incomplete mesentery; MF, mesenterial filament; S, siphonoglyph. Scale bars: 100 µm (*a*,*b*) and 50 µm (*c*).

**Figure 3.**
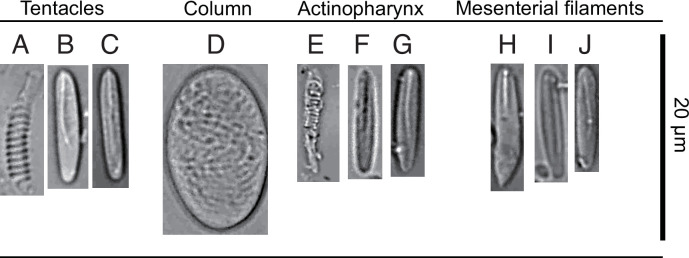
Cnidom in the tentacles, column, actinopharynx and mesenterial filaments of *Corallizoanthus aureus* sp. nov. Capital letters refer to table 1.

**Table 1.** Cnidom types and sizes observed in this study. Frequency: relative abundance of cnidom type in decreasing order; numerous, occasional, rare. *n* = number of cnidom measured. Capital letters in parentheses refer to figure 3.

		*Corallizoanthus aureus* sp. nov.			
tissue	type of cnidae	length (min–max, mean)	width (min–max, mean)	frequency	*n*
tentacle	spirocysts (A)	19.7−14.2, 15.0	2.9−1.7, 2.2	Numerous	57
	basitrichs and microbasic b-mastigophores (B,C)	16.2−10.9, 13.7	3.2−1.9, 2.5	Numerous	98
column	holotrich M (D)	20.9−19.8, 20.1	10.2−12.5, 10.8	Occasional	13
actinopharynx	spirocysts (E)				
	basitrichs and microbasic b-mastigophores (F,G)	14.1−9.8, 11.6	3.5−2.1, 2.9	Rare	3
mesenterial filaments	microbasic p-mastigophores (H)	17.4−9.4, 13.2	4.4−2.9, 3.6	Numerous	69
	basitrichs and microbasic b-mastigophores (I,J)	15.5−13.1, 13.7	3.0−1.9, 2.5	Occasional	13

**Figure 4.**
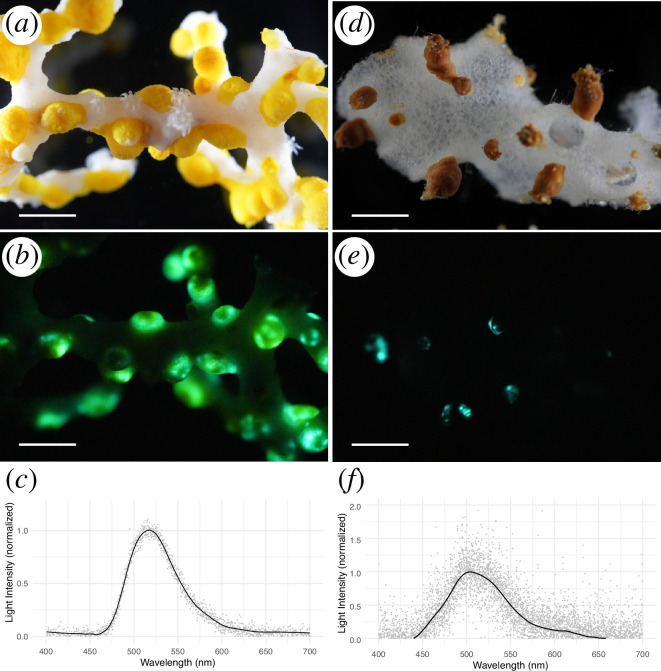
Bioluminescence of *Corallizoanthus aureus* sp. nov. (*a–c*) and *Churabana kuroshioae* (*d*–*f*). The specimen under blight field (*a*), the bioluminescence was triggered by KCl immersion and photographed in the dark (*b*; 2.0 s, f/3.2, ISO 51,200), the representative *in vivo* bioluminescence spectrum with a peak at 515 nm (*c*; FWHM = 67 nm), the specimen under blight field (*d*), the bioluminescence was triggered by KCl immersion and photographed in the dark (*e*; 2.0 sec, f/3.2, ISO 51,200), averaged *in vivo* bioluminescence spectrum (*n* = 4) with a peak at 503 nm (*f*; FWHM = 77 nm). Scale bar 5 mm.

[new Japanese name: uhuagari-akasango-sunaginchaku]

ZooBank: urn:lsid:zoobank.org:pub:93BD8DF6−290C−4662−8CB3-AB2699F2D818

Synonymy. *Corallizoanthus* sp. – Nonaka and Muzik [37] : 24−34, figures 9–12.

Material examined. Holotype: NSMT-Co1915, northwest off Minamidaito Island, Okinawa, Japan, 6 May 2024, 385 m depth. Paratype: NSMT-Co1919, northwest off Minamidaito Island, Okinawa, Japan, 17 July 2025, 385 m depth.

Etymology. The specific name derives from ‘*aureus’* meaning ‘golden’ in Latin.

Diagnosis. Species with 24−26 tentacles, polyps growing over axis and branches of *Pleurocorallium*. Cyclically transitional marginal musculature, actinopharynx unfurrowed.

Description. *External morphology*. Polyps found on all over the surface of *Pleurocorallium inutile* Kishinouye, 1903 [38] (figure 1*a*). Some polyps unitary, others colonial polyps connected by thin stolon (figure 1*c*,*d*). Polyps growing over axis and branches of *P. inutile*. Living polyp, tentacles and oral disk bright yellow (figure 1*b*). Contracted preserved polyps pale yellow in coloration, 2.0−8.0 mm in height, 2.0−5.0 mm in diameter, tentacles and oral disk pale yellow. Tentacles 24−26 in number, capitulary ridges visible 12−13 in number when contracted. Surface of column rough and ectoderm continuous.

*Internal morphology*. Azooxanthellate. Cyclically transitional marginal musculature (figure 1*c*,*d*). Encircling sinus indiscernible and basal canals of mesenteries absent (figure 2*a*). Mesenteries 24−26 in number, in macrocnemic arrangement. Column mesoglea thickness 90−150 µm at level of actinopharynx. Actinopharynx unfurrowed. Siphonoglyph distinct and U-shaped. Mesenterial filaments present. Complete mesenteries infertile (figure 2).

*Cnidom*. Basitrich, holotrich, microbasic p-mastigophore, spirocyst (figure 3, table 1).

Distribution and habitat. *Corallizoanthus aureus* sp. nov. has been found only on *Pleurocorallium inutile*, at depths of 245−400 m, from Tanegashima Island, Kagoshima, Japan [37] and Minamidaito Island, Okinawa, Japan.

Remarks. *Corallizoanthus aureus* sp. nov. can be easily distinguished from its congener species *Corallizoanthus tsukaharai* by the number of tentacles (24–26 versus 18−22) and host octocorals (*Corallium japonicum* Kishinouye, 1903 [38] versus. *Pleurocorallium inutile*) (table 2). Reimer *et al*. [16] found holotrichs and basitrichs in mesenterial filaments in *C. tsukaharai*, while we found microbasic p-mastigophores and basitrichs, but not holotrichs, in mesenterial filaments of *Corallizoanthus aureus* sp. nov. *Corallizoanthus* aff. *tsukaharai* NZ66 and *Corallizoanthus* aff. *tsukaharai* NMNH258 were reported in Swain [39]. However, recent studies have found that these two species belong to *Kulamanamana* and *Hurlizoanthus*, respectively [39,40].

**Table 2.** Comparison of morphological and ecological characters of *Corallizoanthus aureus* sp. nov. and *Corallizoanthus tsukaharai*.

species	distribution	depth (m)	form (colonial or unitary)	sizes of polyps	tentacle and mesentery numbers	cnidom	marginal musculature	reference(s)
*Corallizoanthus tsukaharai*	Ryukyu Archipelago	194−250	unitary	preserved polyp diameter 1.7−3.9 mm, expanded polyp height 12.0 mm	18−22	holotrichs and basitrichs in mesenterial filaments	cyclically transitional marginal musculature	[16,36]
*Corallizoanthus aureus* sp. nov.	Minamidaito Island and Ryukyu Archipelago	245−400	unitary	preserved polyp height 2.0−8.0 mm, diameter 2.0−5.0 mm	24−26	microbasic p-mastigophores and basitrichs in mesenterial filaments	cyclically transitional marginal musculature	[37], this study

**Figure 5.**
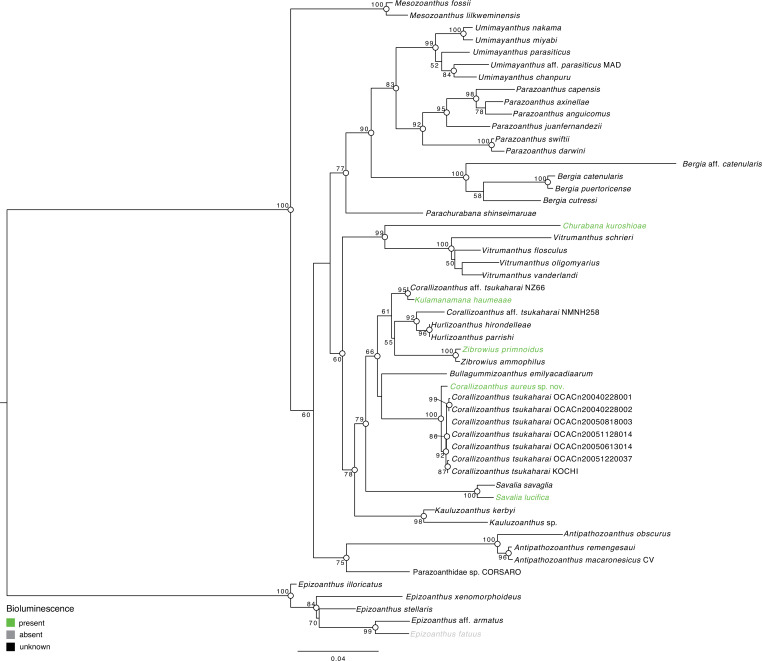
Maximum-likelihood tree based on combined dataset of CoxI, 12S rDNA, 16S rDNA, 18S rDNA, 28S rDNA and ITS rDNA sequences. Numbers at nodes represent ML bootstrap values (>50% are shown). White circles on nodes indicate high support of Bayesian posterior probabilities (PP > 0.95). Green colour indicates bioluminescent species, grey colour indicates non-bioluminescent species and black colour indicates that bioluminescent data were not available.

Molecular phylogeny. Molecular phylogenetic results based on the concatenated dataset indicated that *Corallizoanthus aureus* sp. nov. was sister to *C. tsukaharai* with complete support (ML = 100%, BI = 1) (figure 5).

Mitogenome assembly. The complete mitochondrial genome size of *Corallizoanthus aureus* sp. nov. was 23 098 bp. The mitochondrial gene content was 13 protein-coding genes, two rRNA genes and two transfer RNA genes. GC content was 48.4%. Stop codons were either TAA and TAG for all protein-coding genes, with the start codon being ATG. The mitochondrial base composition was A: 23.4%, T: 28.1%, G: 25.8%, C: 22.7%. The order of the mitochondrial genes followed that previously reported for all other investigated zoantharian species [41].

### 3.2. Bioluminescence activity and spectral properties of emissions

Polyps of *Corallizoanthus aureus* sp. nov. emitted blinking green luminescence upon both mechanical and chemical stimulation, but no luminescence was observed in the stolon (figure 4*a*–*c*, electronic supplementary material, video S1). The emission spectral peak was at 515.4 ± 5.1 nm (4 replicates), with a full width at half maximum (FWHM) of 67.3 ± 2.7 nm. Furthermore, the *in situ* observation of living polyps of *Corallizoanthus aureus* sp. nov. revealed the emission of blinking green luminescence in response to mechanical stimulation, with the luminescence propagating from the stimulation area (electronic supplementary material, video S2). Polyps of *Churabana kuroshioae* also emitted blinking blue-green luminescence, with spectral peak at 503.7 ± 2.0 nm (4 replicates) and a FWHM of 77.0 ± 5.1 nm (figure 4*d–f*, electronic supplementary material, video S3). In contrast, no bioluminescence was observed in *Epizoanthus fatuus*.

## 4. Discussion

To the best of our knowledge, this study represents the first report of bioluminescence observed within a deep-sea cave. In Hexacorallia, bioluminescence has primarily been found in the order Zoantharia, and luminescence has particularly been reported from octocoral-associated zoantharians, including *Epizoanthus induratum* Cutress & Pequenat, 1960 [42] within family Epizoanthidae Delage & Hérouard, 1901 [25], and *Savalia lucifica* (Cutress & Pequenat, 1960) (formally known as *Parazoanthus lucificum*) [42], *Kulamanamana haumeaae* Sinniger, Ocaña & Baco, 2013 [43], *Zibrowius primnoidus* Carreiro-Silva, Braga-Henriques, Sampaio, de Matos, Porteiro and Ocaña, 2010 [44], *Bullagummizoanthus emilyacadiaarum* Sinniger, Ocaña and Baco, 2013 [43] and *Corallizoanthus tsukaharai* [9,43,44] within Parazoanthidae. *Savalia lucifica* has received much attention in bioluminescence studies, and its spectral properties have been described [45]. In contrast, the prevalence of bioluminescence in most other zoantharian species remains largely unknown due to the paucity of proper assessments of luminescence, although luminescence has been simply reported based on field or aquarium observations.

A comprehensive list of bioluminescent species based on accurate identification represents fundamental information for understanding the evolutionary history of bioluminescence. However, zoantharians have been notoriously difficult to identify morphologically due to the small number of measurable characteristics and morphological plasticity [46]. Thus, identification using morphological characteristics alone has led to confusion about the identity of specimens, leading to a lack of accurate data on the presence or absence of luminescent ability in a given species. As with other anthozoan groups, more recent taxonomic assessments of zoantharians have been conducted based on an integrated approach including morphology, molecular phylogeny and ecology (e.g. [17,39,43,47]). The results of these studies have suggested that host specificity could be used to identify epibiotic zoantharians. In particular, the association between Parazoanthidae genera and the host organisms they use as substrates is closely related and phylogenetically constrained [48]. For example, *Corallizoanthus* has only been found on Coralliidae Lamouroux, 1812 [49], including the current *Corallizoanthus aureus* sp. nov. In a previous study, a *Corallizoanthus* associated with *Candidella imbricata* was reported as a bioluminescent species [9], but this specimen likely belongs to *Hurlizoanthus* based on the host octocoral as described in Carreiro-Silva *et al*. [45]. In the current study, *Corallizoanthus aureus* sp. nov. has been formally described, and its bioluminescence has been reported in detail. In addition, we discovered bioluminescence in *Churabana kuroshioae* (Parazoanthidae), a species that is epibiotic on hexasterophoran sponges, but we did not observe any bioluminescence in *Epizoanthus fatuus* (Epizoanthidae), a species living on the stalk of amphidiscosidan sponges. The evolutionary history of bioluminescence in the order Zoantharia is still unclear due to a lack of complete taxon sampling, but we hypothesize that bioluminescence may be a common phenomenon in at least Parazoanthidae based on the combined findings of this study and previous studies [9,43–45]. Further research will help us understand the origin and evolutionary patterns of bioluminescence in Zoantharia.

The chemical basis of bioluminescence in Hexacorallia remains poorly understood. Although it has been suggested that bacteria might be involved [50], no experimental evidence supports this idea to date. In fact, our histological observations revealed no indications of symbiotic bacterial cells (figure 2). An alternative hypothesis posits that hexacorals utilize an intrinsic bioluminescent system involving a chemical substrate and an enzyme, known as luciferin and luciferase, respectively [51]. Coelenterazine, a luciferin commonly found in various marine bioluminescent organisms—including several cnidarian lineages such as Malacalcyonacea, Hydromedusae and to a lesser extent, Scyphozoa—has also been detected in hexacorals, including *Savalia lucifica* and an unidentified Hormathiidae species [1,5,8,9,52,53]. Coelenterazine-dependent luciferase activity has been experimentally demonstrated in both *S. lucifica* and Hormathiidae sp. [5,54]. However, biochemical and evolutionary analyses suggest that the type of luciferase involved in these species may differ from that found in Malacalcyonacean octocorals [5,9,54]. Thus, it seems like coelenterazine-dependent luciferase is involved in the bioluminescence in *Corallizoanthus aureus* sp. nov. and *C. kuroshioae*, although further experimental verification is required.

The bioluminescence of *Corallizoanthus aureus* sp. nov. and *Churabana kuroshioae* was green (peak wavelength = 515 nm) and blue-green (peak wavelength = 504 nm), respectively (figure 4). The green bioluminescence of *S. lucifica* has also been reported, with a peak at 509−520 nm [45,55]. Interestingly, some polyps within a single colony of *S. lucifica* have been observed to emit light at longer wavelengths, with a peak at 574 nm. In our study, we did not observe multicolor bioluminescence within any single species. Under UV light (wavelength = 365 nm), neither *Corallizoanthus aureus* sp. nov. nor *Churabana kuroshioae* showed fluorescence, suggesting that fluorescent proteins are not involved in the bioluminescence in these species. Colour variation among intraspecies or interspecies may be attributable to differences in the amino acid sequences of the luciferases. For example, genetically engineered luciferase of *Renilla reniformis* (Octocorallia) showed an approximately 60 nm spectral shift in emission wavelength (from 480 nm to 540 nm) following 16 amino acid substitutions [56,57]. Therefore, identification and functional characterization of the luciferase enzymes in Parazoanthidae hexacorals may provide critical insights into the molecular basis of bioluminescent colour variation within this group.

This study tested the bioluminescence of *Corallizoanthus aureus* sp. nov. via stimulation, both on board and *in situ*. The two observations yielded the same result, finding blinking green luminescence upon mechanical stimulation. However, spontaneous luminescence was not observed in this study. Such behaviour indicates that bioluminescence in those species plays defensive roles. Although the ecological function of bioluminescence in Zoantharia is not known, it may act as a ‘burglar alarm’ as suggested by Burkenroad [58], in which bioluminescence emitted by the prey attracts second-order predators to attack the first-order predators [59]. Another ecological function might be a luminous sacrificing tag. The sticky glowing mucus or autotomized body parts attach onto the predator, and it may help to temporarily make the predators visible and vulnerable against higher predators, and this could entice enemies (predators) of such predators. A similar hypothesis was suggested for the deep-sea cucumber *Enypniastes eximia* Théel, 1882 [60,61]. With the growing number of reports documenting bioluminescence, especially among hexacorals where commensal relationships are frequent [9,44,45], bioluminescence may serve as a means of communication among commensal organisms, although further investigation is clearly needed. Deep-sea zoantharians are typically in symbioses with other invertebrates such as anthozoans, sponges, decapods and molluscs (e.g. [16,39,40,43,48]), making Zoantharia a potential exemplar taxon to study bioluminescence. Thus, integrating bioluminescence information into zoantharian studies would allow us to understand not only evolutionary but also the ecological aspects of bioluminescence in deep-sea environments.

## Data Availability

The videos showing bioluminescence are appended as supplemental information [62]. Obtained sequences and mitogenome have been deposited in NCBI GenBank (PV930384– PV930386 and PV987613, respectively).
